# Sugar-Binding Profiles of Chitin-Binding Lectins from the Hevein Family: A Comprehensive Study

**DOI:** 10.3390/ijms18061160

**Published:** 2017-05-30

**Authors:** Yoko Itakura, Sachiko Nakamura-Tsuruta, Junko Kominami, Hiroaki Tateno, Jun Hirabayashi

**Affiliations:** 1 Biotechnology Research Institute for Drug Discovery, National Institute of Advanced Industrial Science and Technology, AIST Tsukuba Central 2, 1-1-1, Umezono, Tsukuba, Ibaraki 305-8568, Japan; yitakura@tmig.or.jp (Y.I.); stsuruta@live.minatogawa.ac.jp (S.N.-T.); juncommy@gmail.com (J.K.); h-tateno@aist.go.jp (H.T.); 2 Tokyo Metropolitan Institute of Gerontology, 35-2 Sakae-cho, Itabashi-ku, Tokyo 173-0015, Japan

**Keywords:** chitin-binding lectin, hevein domain, frontal affinity chromatography, sugar-binding specificity, microarray

## Abstract

Chitin-binding lectins form the hevein family in plants, which are defined by the presence of single or multiple structurally conserved GlcNAc (*N*-acetylglucosamine)-binding domains. Although they have been used as probes for chito-oligosaccharides, their detailed specificities remain to be investigated. In this study, we analyzed six chitin-binding lectins, DSA, LEL, PWM, STL, UDA, and WGA, by quantitative frontal affinity chromatography. Some novel features were evident: WGA showed almost comparable affinity for pyridylaminated chitotriose and chitotetraose, while LEL and UDA showed much weaker affinity, and DSA, PWM, and STL had no substantial affinity for the former. WGA showed selective affinity for hybrid-type *N*-glycans harboring a bisecting GlcNAc residue. UDA showed extensive binding to high-mannose type *N*-glycans, with affinity increasing with the number of Man residues. DSA showed the highest affinity for highly branched *N*-glycans consisting of type II LacNAc (*N*-acetyllactosamine). Further, multivalent features of these lectins were investigated by using glycoconjugate and lectin microarrays. The lectins showed substantial binding to immobilized LacNAc as well as chito-oligosaccharides, although the extents to which they bound varied among them. WGA showed strong binding to heavily sialylated glycoproteins. The above observations will help interpret lectin-glycoprotein interactions in histochemical studies and glyco-biomarker investigations.

## 1. Introduction

Historically, plant lectins have been characterized as hemagglutinin- or mitogen-acting toward animal cells (e.g., *Griffonia simplicifolia* lectin I (GSL-I) A4 hemagglutinin specific for blood type group A [1,2,3] and the plant toxin ricin known as *Ricinus communis* agglutinin II (RCA60) [4,5,6]). Plant lectins exist as multiple molecular species (i.e., isolectins such as *Ricinus communis* agglutinin I (RCA120), RCA60, GSL-I A4, and GSL-I B4 [1,7,8,9,10,11]). These plant lectins have long been utilized in the glycoscience field, especially when no methods are available other than anti-glycan antibodies. Therefore, knowledge about glycan-binding properties is crucial to interpret the results obtained by their use.

**Figure 1:**
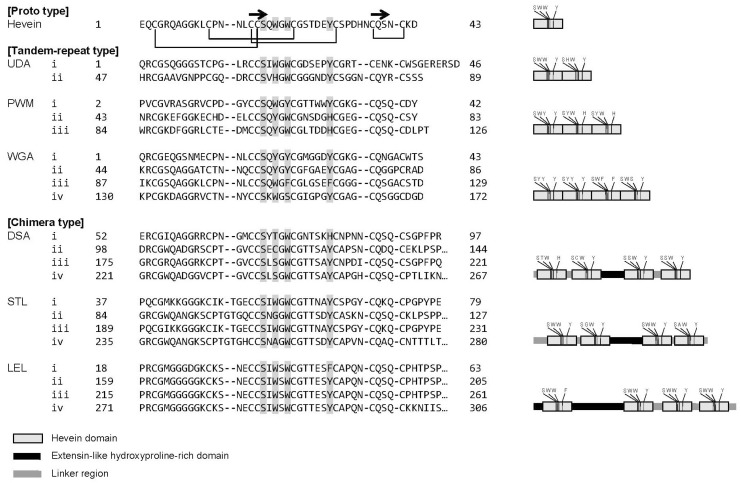
Alignment of amino-acid sequences of the six chitin-binding lectins investigated in this work. Hevein, constituting a sole proto-type lectin, is shown as a reference. The sequences are of UDA6 isolectin (GenBank/EMBL/DDBJ accession number AAD05433) for UDA, PLC isolectin (AB052963) for PWM, WGA1 isolectin (UniProtKB accession number, P10968) for WGA (categorized as tandem-repeat type), LEL (AB360604), DSA-B isolectins (AB618634) for DSA, and STL (Q9S8M0; categorized as chimera type). Bold arrows on the top denote the regions of β-strands. Schematic representations are made for each lectin on the right, where four amino-acid residues essential for chito-oligosaccharide-binding in hevein, i.e., Ser (S), two Trp (W), and Tyr (Y) are shown in each hevein domain shown with a gray box. The extensin-like, function-unknown domain is shown with a black bar.

Chitin-binding lectins constitute members of the hevein-like lectin family (hereafter referred to as the hevein family), where hevein corresponds to a sole proto-type member of the family, consisting of 43 amino acids (hevein domain), including evolutionarily conserved serine and aromatic residues as well as disulfide bridges (Figure 1). Hevein was originally found in latex from rubber as a component of chitinase in 1970. Although hevein is known to show relatively weak (i.e., 1 mM or lower *K*_d_) affinity for chito-oligosaccharides [12,13,14], other chitin-binding lectins have multiple carbohydrate-binding domains and their amino acid sequences are significantly different, including the conserved aromatic residues involved in GlcNAc recognition (Figure 1).

Van Damme has proposed the categorization of the known plant lectins, including chitin-binding lectins, into three groups: merolectin, horolectin, and chimerolectin. Further, superlectin was designated as a special group of chimerolectin [15]. This classification is analogous to that of galectins forming a large group of animal lectins (e.g., “proto type” consisting of a single carbohydrate-recognition domain (CRD) such as galectin-1, 2 and 7, “tandem-repeat type” consisting of multiple CRDs such as galectin-4, 8 and 9, and “chimera type” consisting of a CRD and a non-CRD structural domain such as galectin-3 [16]). With regard to the binding specificity of chitin-binding lectins, WGA is known to bind heavily sialylated glycans [17,18] and tomato lectin (LEL) preferentially binds to repeated structures of *N*-acetyllactosamine (poly-LacNAc) [19,20]. However, details of the glycan-binding specificity of these chitin-binding lectins are not known, even though they are well-known plant lectins.

In this study, six chitin-binding lectins, *Datura stramonium* agglutinin (DSA), *Lycopersicon esculentum* (currently renamed *Solanum lycopersicum*) lectin (LEL; tomato lectin), *Phytolacca americana* mitogen (PWM; pokeweed mitogen), *Solanum tuberosum* lectin (STL; potato lectin), *Urtica dioica* agglutinin (UDA), and *Triticum vulgaris* agglutinin (WGA; wheat germ agglutinin), belonging to the hevein family, were investigated for their glycan-binding properties. WGA is extensively used as a probe to stain cell membranes [21,22]. LEL is also used for histochemical staining of the vascular endothelium [23,24]. Other lectins are also used as probes to stain animal tissue sections [25,26]. WGA isolated from the germ of wheat (Gramineae) is a tandem-repeat type lectin consisting of four hevein domains of homo or hetero dimers [27,28,29,30,31,32]. As shown by Yamaguchi et al. and Hayashida et al. [14,33,34,35], PWM isolated from the root of pokeweed (Phytolaccaceae) also belongs to a tandem-repeat type, existing as a dimer. UDA isolated from the rhizome of stinging nettle (Urticaceae) is also a tandem-repeat type lectin, consisting of two hevein domains [36,37,38]. In contrast, STL isolated from the plant tuber potato and DSA isolated from the seed of thornapple (Solanaceae) are more complex and categorized as a chimera-type lectin, both of which contain four hevein domain repeats and an extensin-like domain, the latter of which is located between two N-terminal hevein domains and two C-terminal domains, the function of which is not known [39,40,41,42,43]. LEL isolated from the fruit of tomato (Solanaceae) also belongs to a chimera-type lectin, comprised of a single and three hevein domain repeats in the N- and C-terminus of the dimer, respectively, and an extensin-like domain in between, unlike STL and DSA [44,45] (Figure 1).

We analyzed the glycan-binding properties of the six chitin-binding lectins for 124 pyridylaminated (PA) oligosaccharides by quantitative frontal affinity chromatography (FAC) [46,47,48], where lectins are immobilized. Comparative analysis of the six lectins were also conducted using a glycoconjugate microarray, where a series of glycoconjugates consisting of glycoproteins and synthetic glycans conjugated to polyacrylamide (PAA), are immobilized on a glass slide [49]. Further analysis by a lectin microarray, where 43 commercial lectins, including the six chitin-binding lectins, are immobilized [50,51], was performed by probing with a serum glycoprotein, fetuin (FET), both before and after sialidase treatment to examine how the signal patterns are changed on the chitin-binding lectins. The observed results are extremely informative and, thus, they will help interpret lectin-glycoprotein interactions in a variety of histochemical research fields and glyco-biomarker investigations.

## 2. Results and Discussion

### 2.1. A Strategy for Comprehensive Analysis of Sugar-Binding Properties of Chitin-Binding Lectins

The overall strategy in this work of analyzing six chitin-binding lectins used the following three approaches: (1) binding affinities between the six lectins and a panel of PA-oligosaccharides were determined in terms of dissociation constant (*K*_d_) by FAC and based on the FAC results; (2) their actual binding to immobilized glycans was analyzed using a glycoconjugate microarray, wherein 96 glycoconjugates consisting of serially glycosidase-treated glycoproteins and polymer-based multivalent synthetic glycans were immobilized; and (3) effects of desialylation were examined with a model serum glycoprotein, FET, on a lectin microarray platform, wherein 43 commercial lectins, including the six chitin-binding lectins, were immobilized.

### 2.2. FAC for Determination of K_d_’s to a Panel of PA-Oligosaccharides

For systematic comparison of sugar-binding properties of the six chitin-binding lectins, FAC was carried out with a previously established procedure [9,48,52]. Briefly, to determine the *K*_d_ values of each of the six chitin-binding lectins for 124 PA-oligosaccharides, each of the lectins was immobilized on activated Sepharose at several concentrations: one for concentration-dependent analysis to determine the effective ligand content *B*_t_ (expressed in nmol) of a standard column, from which *K*_d_ values for a panel of PA-oligosaccharides were determined according to a simplified basic equation of FAC, *K*_d_ = *B*_t_/(*V* − *V*_0_). The other(s) are supplementary to cover a wide range of possible *K*_d_ values, which is in direct relation to the observed value of *V* − *V*_0_ (for more detail, see Materials and Methods).

**Figure 2:**
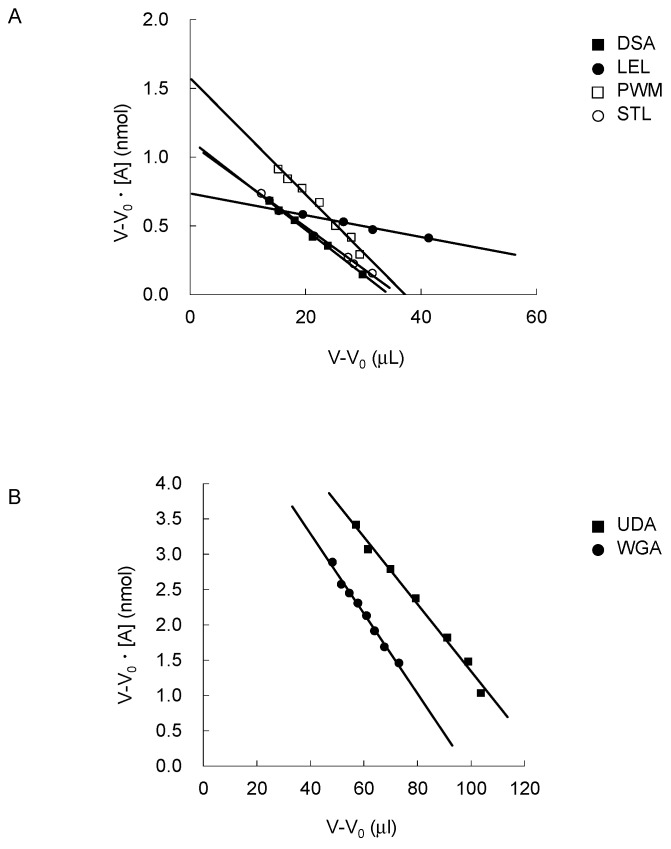
Woolf-Hofstee-type plots for the determination of *B*_t_ values. The results of columns for DSA (closed squares), LEL (closed circles), PWM (open squares) and STL (open circles), and those for UDA (closed squares) and WGA (closed circles) are shown in panels A and B, respectively. For concentration-dependent analysis, appropriate *p*NP-sugars were used: LacNAc-β-*p*NP for LEL, UDA, and WGA, chitotetraose-β-*p*NP for DSA, and chitopentaose-β-*p*NP for PWM and STL columns. Plots were made by using data, *V* − *V*_0_ vs. (*V* − *V*_0_) [A]_0_, obtained in the concentration range 2–60 μM. For details, see text.

In this work, *B*_t_ was determined for each column using an appropriate *p*-nitrophenyl (*p*NP)-saccharide, which showed significant affinity with sufficient retardations (>25 μL). Concentration-dependent analysis showed that *B*_t_ values were 1.12, 0.74, 0.11, 1.58, 1.10, 6.10, and 5.55 nmol for DSA, LEL, LEL (supplementary), PWM, STL, UDA, and WGA columns, respectively (Table 1). From these data, *K*_d_ values were derived to be 33 μM for DSA and chitotetraose-β-*p*NP, 7.9 μM for LEL and LacNAc-β-*p*NP, 4.6 μM for LEL and chitopentaose-β-*p*NP, 42 μM for PWM and chitopentaose-β-*p*NP, 31 μM for STL and chitopentaose-β-*p*NP, 48 μM for UDA and LacNAc-β-*p*NP and 57 μM for WGA and LacNAc-β-*p*NP (Figure 2).

### 2.3. Global Features of Sugar-Binding Properties of Chitin-Binding Lectins

**Figure 3:**
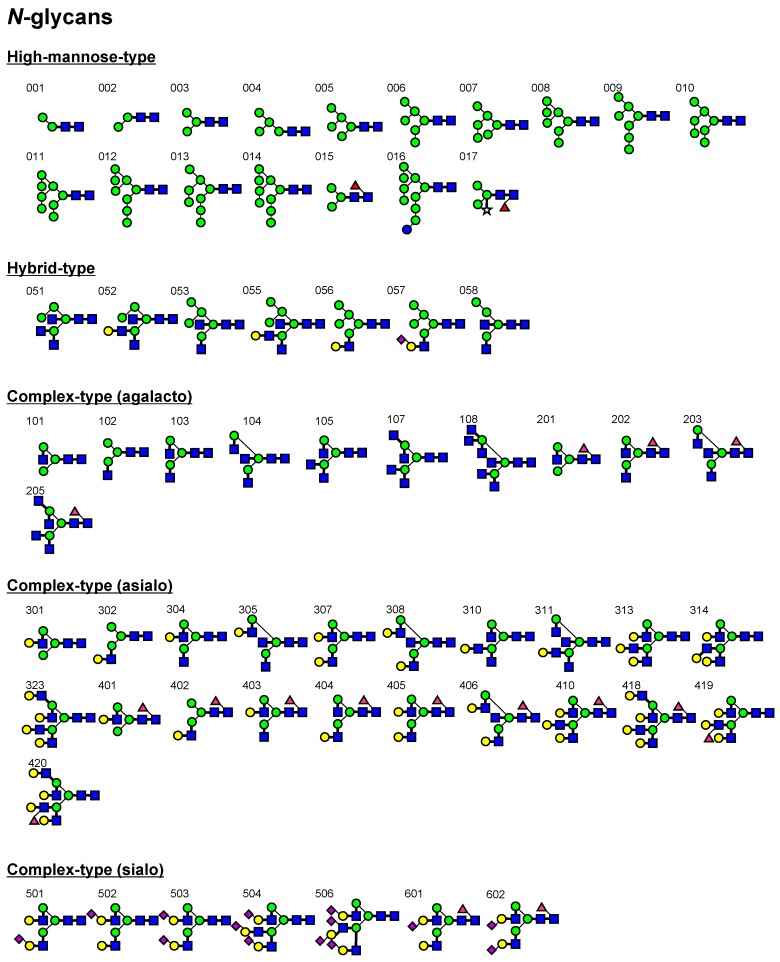

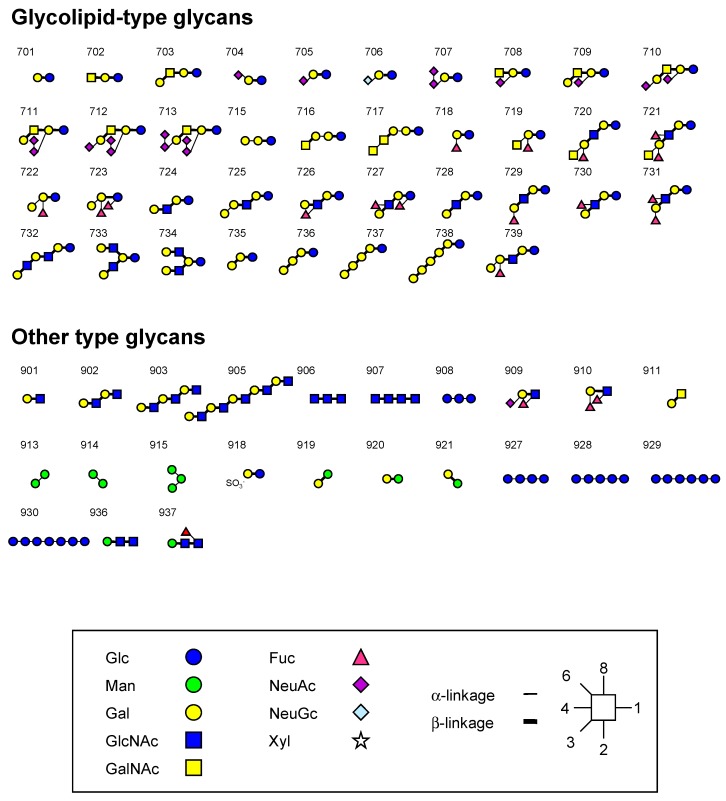
A list of PA-oligosaccharide structures used in this work. Note that the reducing terminal of each PA-oligosaccharide is in an open form because of monoamine coupling with 2-aminopyridine. Carbohydrate structures are expressed using symbols according to the rules defined by the Consortium for Glycomics (CFG; http://www.functionalglycomics.org/static/consortium/Nomenclature.shtml) with further modification to specify linkage and anomeric types: position 1, is placed at the right side, and numbering proceeds clockwise. Thin and thick bars represent α- and β-linkages, respectively.

**Figure 4:**
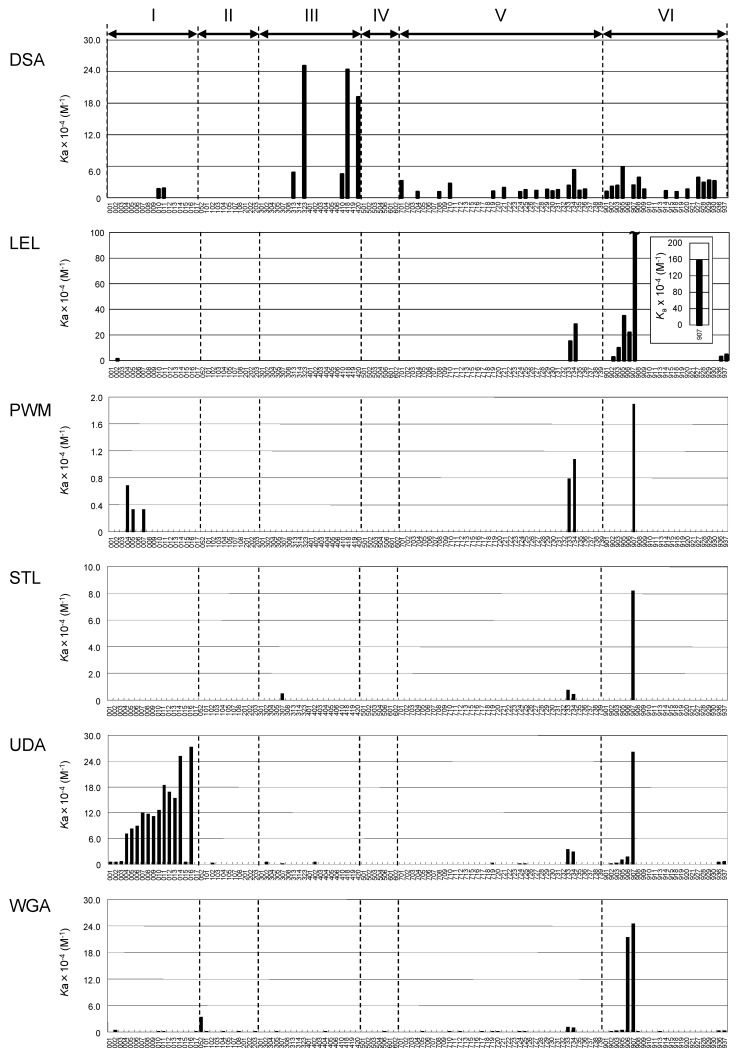
Bar graph representation of affinity constant (*K*_a_) was made for DSA, LEL, PWM, STL, UDA, and WGA against a panel of PA-oligosaccharides. The small Arabic numbers at the bottom of each graph correspond to sugar numbers indicated in Figure 3, whereas the Roman numerals at the top represent types of glycans, i.e., high-mannose-type (I), agalacto-type (II), asialo-type (III), sialo-type (IV) *N*-glycans, glycolipid-type glycans (V), and others (VI). For oligosaccharide structures and specifications of the lectin columns, see Figure 3 and Table 1, respectively. *K*_d_ values obtained in this analysis are listed in Table S1.

Based on the above results, *K*_d_ values were subsequently determined for a panel of PA-oligosaccharides (Figure 3). Hereafter, FAC data are expressed in terms of affinity constant *K*_a_ (1/*K*_d_) in the bar graphs in Figure 4, while they are discussed in terms of *K*_d_ (μM) throughout the text (also see Table S1).

As has been previously reported in studies of chitin-binding lectins [40,53,54,55,56,57,58,59,60], LEL, PWM, STL, UDA, and WGA showed high affinity, while DSA showed low affinity for chito-oligosaccharides (indicated by numbers **906** and **907** in the figures). However, the determined *K*_d_ values varied considerably among the six lectins. Those determined for PA-chitotetrasaccharide (**907**; note that reducing terminal GlcNAc takes an open form as a result of pyridylamination and, thus, may not contribute to full recognition in some lectins) were 43 (DSA), 0.64 (LEL), 53 (PWM), 12 (STL), 3.8 UDA), and 4.1 μM (WGA). On the other hand, DSA, PWM, and STL did not show detectable affinity for chitotriose (**906**), while the *K*_d_ value of WGA was almost the same as that for chitotetraose. Notably, observed affinities of DSA for these chito-oligosaccharides were relatively low compared with other oligosaccharides to which DSA binds, such as branched *N*-glycans (**323** and **418**). In this context, DSA is unique in that it showed significant affinity for a series of oligo-α1-4-glucose, (Glcα1-4)_3__–7_ (**908**, **927**–**930**). However, their affinities were almost the same regardless of the chain length, unlike other oligomers (described in detail below).

### 2.4. Depicted Features of Individual Lectins

#### 2.4.1. DSA

DSA affinity was higher for type II than type I structures on both *N*-glycans: **313** (*K*_d_ 21 μM) > **314 **(not detectable, hereafter referred to as N.D.) and glycolipid-type glycans **724** (91 μM) > **728 **(N.D.), and **734** (19 μM) > **733** (43 μM). It was also evident that Lewis-type fucosylation abolished DSA binding to type II LacNAc by comparison between triantennary **313** and **419** (N.D.), and between tetraantennary **323** (4.0 μM) and **420** (5.2 μM), though the effect of Le^x^ inclusion was less dominant in the latter. This feature was more evident for glycolipid-type glycans **724** (L*N*nT, 91 μM) vs. **726** (Le^x^, N.D.). DSA affinity appeared to be attributed to the number of intact type II LacNAc units.

Conversely, core (α1-6) fucosylation did not substantially affect the affinity (**410**, 22 μM and **418**, 4.1 μM compared with **313,** 21 μM and **323**, 4.0 μM, respectively). This result clearly indicates that DSA does not bind to the non-reducing terminal chitobiose structure, likely because the structure does not fulfill the minimal requirement for DSA recognition. Though the affinity is relatively low, DSA showed selective binding to high-mannose-type *N*-glycans **010** (60 μM) and **011** (56 μM).

#### 2.4.2. LEL

As has already been reported, both DSA and LEL show significant affinity for poly-LacNAc [19,20,61,62,63]. However, as noted above, LEL showed much stronger affinity for both chito-oligosaccharides (**906** and **907**) and oligo-LacNAc (**901**, **902**, **903**, and **905**) than DSA. When the affinities of LEL for **901**, **902**, **903**, and **905** were compared, it was evident that LEL affinity increased with the increasing number of LacNAc units: **901** (N.D.) < **902** (39 μM) < **903** (10 μM) < **905 **(2.9 μM). DSA showed a similar tendency, but affinities to these oligo-LacNAc structures are relatively low; i.e., **901** (87 μM) < **902** (48 μM) < **903** (43 μM) < **905 **(17 μM). Thus, LEL appears to work as a good probe for detecting oligo- or poly-LacNAc chains in *N*-, *O*-glycans, or glycolipids. On the other hand, affinities for these oligosaccharides were low for WGA and UDA, and not detectable for PWM and STL, though previous papers report that PWM bound to poly-LacNAc chains with a GlcNAcβ1-6Gal branched structure, which is the result of I-GlcNAc transferase action [64].

Like DSA, LEL showed an apparent preference for type II LacNAc structure by comparison of branched glycolipid-type structures, i.e., **733** (6.7 μM) < **734** (3.5 μM). Intriguingly, the lectin did not bind to other branched glycans such as **323** and **418**, to which DSA has the highest binding affinity. Therefore, their recognition mechanisms substantially differ from each other at a molecular level.

#### 2.4.3. STL

Among the chitin-binding lectins tested, STL showed the simplest profile of high affinity to only chitotetraose (**907**, 12 μM). Significant binding was also found for other oligosaccharides, **307** (210 μM), **733** (130 μM), and **734** (220 μM), although the affinities were relatively low. When type I and type II LacNAc structures were compared; i.e., **733** vs. **734**, affinities for these glycolipids-type oligosaccharides were almost the same, with affinity for type I being slightly higher. As is described later, STL showed selective affinity for a LacdiNAc (GalNAcβ1-4GlcNAc) structure when analyzed by glycoconjugate microarray, although the FAC assay did not generate corroborating evidence for this because of the lack of appropriate PA-oligosaccharides.

#### 2.4.4. PWM

As shown in Figure 4 and Table S1, binding affinity of PWM is lowest among the tested chitin-binding lectins. In fact, the *K*_d_ for PA-chitotetraose (**907**), for which PWM showed the highest affinity, is 53 μM, which is 83 times weaker than LEL. However, PWM showed substantial affinity for glycolipid-type glycans **733** and **734**. As found in the case for DSA and LEL, PWM showed preference for type II structure (**734**, 93 μM) over type I (**733**, 130 μM).

Another novel finding is that PWM showed significant affinity for high-mannose-type *N*-glycans **004** (150 μM), **005** (300 μM), and **007** (300 μM). This result suggests that PWM can bind glycans with elongation of the Manα1-6Manβ branch, but α1-2 extension from the α1-3Manβ branch blocks the binding. This observation conflicts with a previous report that PWM (Pa-2) binds glycans with Manα1-2 extension of the α1-3Manβ branch [55]. This discrepancy may be ascribed to the occurrence of isolectins, because the PWM used in this study was commercial product [65]. In this context, it is intriguing to know which feature is functionally related to the mitogenic activity of PWM and DSA in terms of isolectins, which other chitin-binding lectins lack [66,67].

#### 2.4.5. UDA

**Figure 5:**
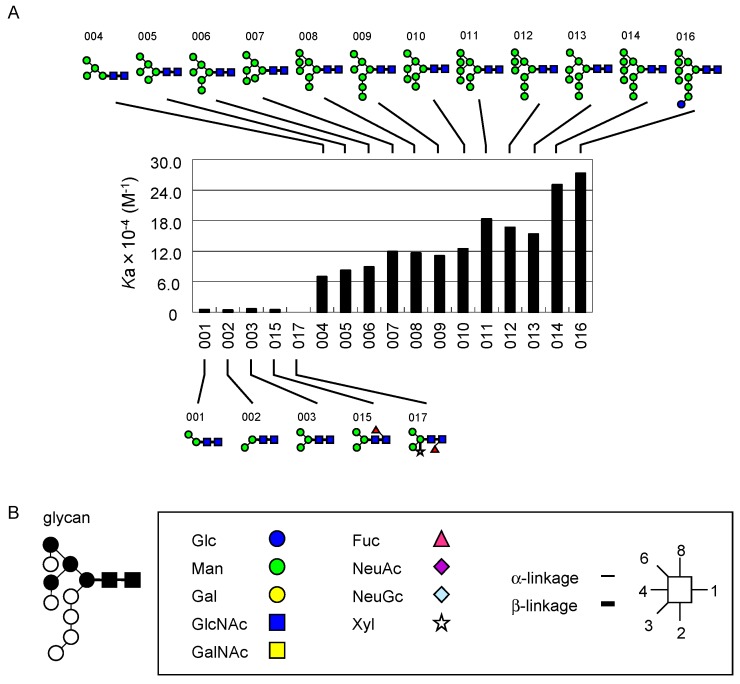
Featured specificity of UDA for high-mannose-type glycans. Bar graph representation for affinity constant (*K*_a_) in relation to the number of mannose residues (**A**); Bound and unbound (or weakly bound) glycan structures are shown at the top and bottom of the graph, respectively. Schematic drawings of a high-mannose structure, where essential and non-essential mannose residues are shown in closed and open circles, respectively (**B**). For carbohydrate structures using symbols, see explanation in the box below as in Figure 3.

In the present work, it was found that UDA bound to a series of high-mannose-type *N*-glycans (**001**–**014** and **016**), although there are only a few reports on such interactions [68]. In the present study, however, it became evident that the affinity to these glycans increased with the increasing number of Man residues (Figure 5A). Detailed structural inspection leads to speculation that UDA requires the presence of a Manα1-3(Manα1-6)Manα1-6Manβ structure, in which both non-reducing terminal Manα1-3 and Manα1-6 can be modified with Manα1-2 (Figure 5B). This is significantly different from PWM, which does not permit α1-2Man extension from the Manα1-3Manβ branch in the common recognition unit Manα1-3(Manα1-6)Manα1-6Manβ. These findings provide clues to interpret the results of binding experiments using these lectins as probes for high-mannose-type *N*-glycans.

#### 2.4.6. WGA

It is widely known that WGA interacts with heavily sialylated glycans on glycoproteins making a sialic acid cluster [17,18,69]. However, using FAC analysis, WGA showed no apparent affinity for sialoglycans (**501**–**602**), with the sole exception being **506** (930 μM), a hypersialylated triantennary *N*-glycan. Although the binding is marginal, the retardation is significant in terms of *V* − *V*_0_ (6 μL). Marginal but significant binding was also observed for sialylated glycans **705** (1000 μM), **710** (1000 μM), and **712** (1100 μM), which contain Neu5Acα2-3Gal. In fact, WGA is extensively used as a practical probe for glycoproteins with α2-3Sia rather than with α2-6Sia.

**Figure 6:**
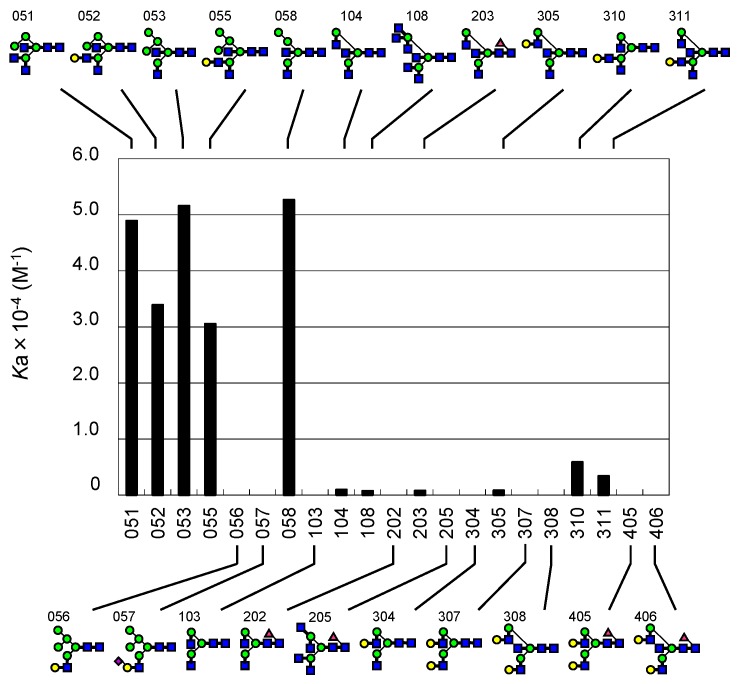
Featured specificity of WGA for hybrid-type *N*-glycans containing bisecting GlcNAc. A bar graph of affinity constant (*K*_a_) in relation to the presence or absence of bisecting GlcNAc was made. Bound and non-bound glycan structures are shown at the top and the bottom of the graph, respectively.

In our initial round of FAC analysis using a standard set of PA-oligosaccharides, significant binding was found for **052** (29 μM), a sole hybrid-type *N*-glycan with a bisecting GlcNAc residue. Yamamoto et al. previously reported that WGA bound to hybrid-type *N*-glycans [70]. Therefore, we undertook further analysis using an additional set of hybrid-type *N*-glycans, i.e., **051**, **053**, **055**, **056**, **057**, and **058**. We found that WGA specifically bound to hybrid-type *N*-glycans with a bisecting GlcNAc residue (**051**, **052**, **053**, **055**, and **058**), but did not bind to molecules without it (**056** and **057**, Figure 6). However, WGA showed much lowered binding to complex-type *N*-glycans, even though they are equipped with a bisecting GlcNAc residue, **104** (1000 μM), **108** (1400 μM), **203** (1200 μM), **305** (1200 μM), and **311** (290 μM). The affinity significantly decreased with the introduction of a LacNAc structure with GlcNAc transferase (GnT) IV, as seen in comparisons between **051** (20 μM) and **052** (29 μM) and between **053** (19 μM) and **055** (33 μM). A sole exception is **310** (170 μM), which is a GlcNAc-exposed triantennary complex-type *N*-glycan lacking a bisecting GlcNAc residue. Considering the fact that WGA has no binding affinity for fully galactosylated glycans **308** and **406**, which contain a bisecting GlcNAc residue, nor for an agalactosylated biantennary glycan **103**, substantial binding to **310** as well as its analogue **311** could be attributed to the occurrence of a non-reducing terminal GlcNAc at two branch positions caused by GnT I and II as well as branching catalyzed by GnT IV.

### 2.5. Specificity for Multivalent Oligosaccharides: Binding Features on Glycoconjugate Microarray

The above FAC analyses provided basic data in terms of *K*_d_ observed between immobilized lectins and fluorescently labeled diluted glycans. Lectins often exert much stronger binding (or avidity) toward multivalent forms of glycans, termed as the “clustering effect of glycosides” [71]. To investigate the affinity for multivalent oligosaccharides, the binding of the six chitin-binding lectins to immobilized glycans either in form of glycoproteins or synthetic glycans chemically conjugated with PAA was analyzed using a glycoconjugate microarray [49]. For reference, related glycans used in FAC are indicated in parentheses.

#### 2.5.1. Binding Features to Synthetic Glycans Conjugated to PAA

**Figure 7:**
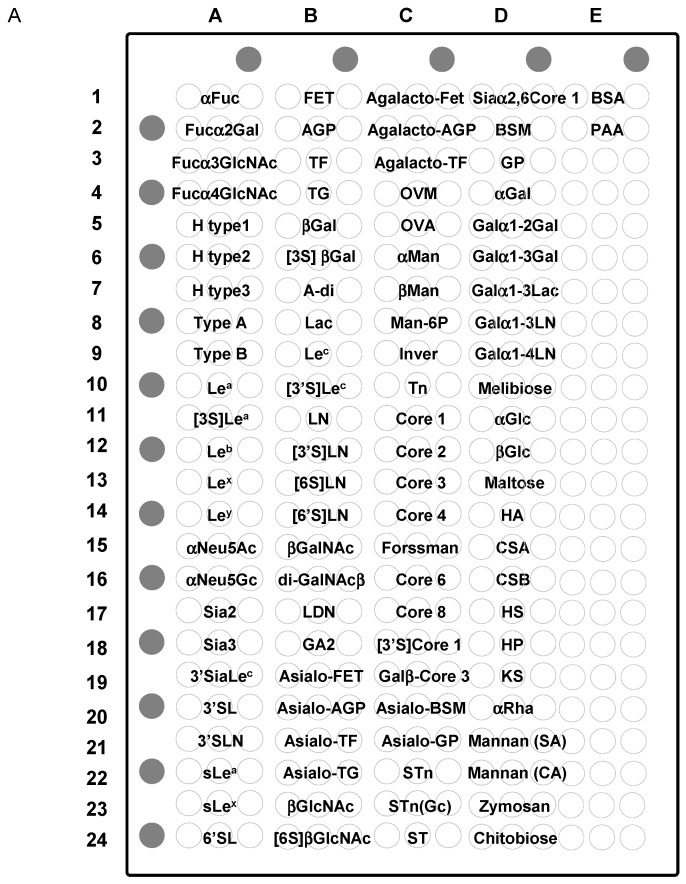

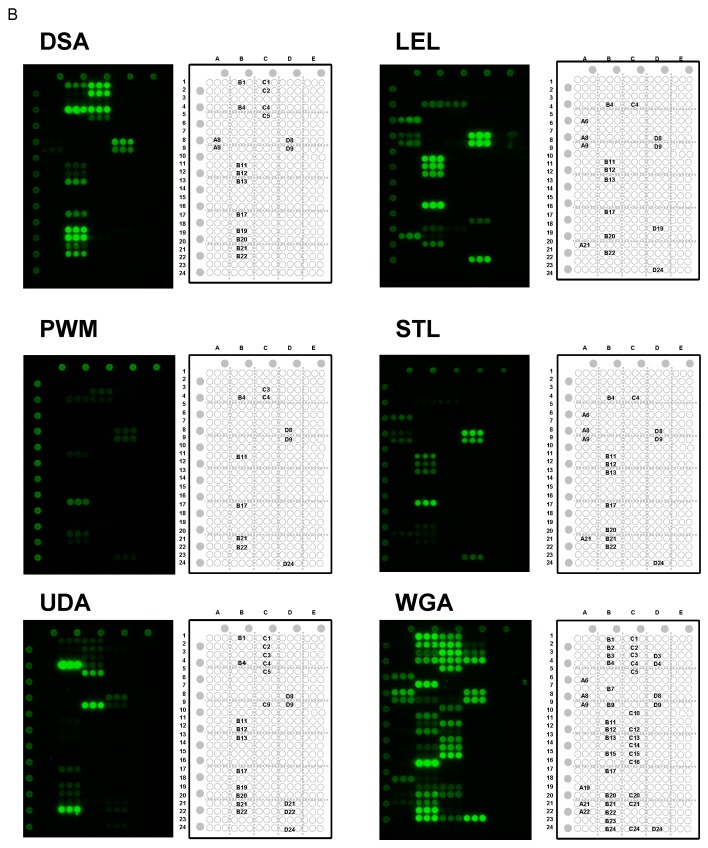
Glycoconjugate microarray analysis to evaluate binding to multivalent glycans. Spot pattern of the glycoconjugate microarray (ver.4.2), where 96 synthetic glycan-conjugated polyacrylamides (PAAs) and glycoproteins were immobilized to a glass slide in triplicate (**A**); BSA (bovine serum albumin) and PAA were included as negative controls. Position markers (Cy3-BSA) were spotted and are depicted as grey-colored closed circle. Signal patterns obtained for each of Cy3-labeled chitin-binding lectins are shown (**B**). For reference, positive signals are abbreviated in the right panel of each fluorescence image.

Although only LEL and WGA showed significant affinity for PA-chitotriose in the FAC analysis, STL, PWM, and UDA also showed significant binding to chitobiose-PAA (**D24**; **906** in Figure 3) on the microarray platform, though their binding was much weaker than LEL and WGA (Figure 7; for bar graph representation see Figure S1). However, DSA binding was not detected. Overall, binding affinities to the immobilized glycans were enhanced in this assay system.

All six chitin-binding lectins showed significant binding to LacNAc (LN; **B11**; **901** in Figure 3) and its derivatives Galα1-3LacNAc (**D8**; **725** in Figure 3) and Galα1-4LacNAc (**D9**; **715** in Figure 3), although the binding features were different from one another. The binding to LacNAc-PAA (**B11**) was apparently strong for LEL and STL, while it was low for PWM, UDA, and DSA. LEL and STL bound almost equally well to sulfated LacNAc-PAA, [3’S]LacNAc (**B12**; **918** in Figure 3), and [6S]LacNAc (**B13**; not available in Figure 3), whereas WGA and DSA showed preference for [6S]LacNAc (**B13**) over [3’S]LacNAc (**B12**). The binding to Galα1-3LacNAc and Galα1-4LacNAc is not ascribed to terminal epitope structures (e.g., αGal epitope), because such terminal disaccharides (Galα1-3Gal and Galα1-4Gal) were not recognized at all (Figure 7).

As unique features to individual lectins, only LEL bound to keratin sulfate (KS; **D19**) but not to other glycosaminoglycans such as hyaluronic acid (HA; **D14**), chondroitin sulfate A (CSA; **D15**), chondroitin sulfate B (CSB; **D16**), heparin sulfate (HS; **D17**), and heparin (HP; **D18**). Although it has been reported that STL binds to KS [72,73], STL did not show the binding to KS (**D19**) in this study. Considering that [6S]LacNAc, as well as its precursor LacNAc, are major components of KS, the inability of STL to bind to this neoglycoprotein (KS chemically conjugated to bovine serum albumin [49]) is possibly a result of insufficient interaction with the epitope structures under the experimental conditions employed in this work.

All six chitin-binding lectins showed substantial binding to LacdiNAc (**B17**; not available in Figure 3), while no binding was observed to Lac (**B8**; **701** in Figure 3), indicating the importance of the presence of the lateral acetyl group of LacdiNAc unit. However, the binding was particularly evident in LEL, STL, and WGA. Notably, STL binding to this disaccharide was more selective than the other lectins. Considering that Galα1-3LacNAc, to which STL bound most strongly, is a xeno-antigen, and thus absent in human-derived samples, it may be a good probe for LacdiNAc structures in clinical samples in place of conventional *Wisteria floribunda* agglutinin (WFA), which binds to both α- and β-GalNAc [74]. WFA also binds strongly to glycoproteins, which have highly branched *N*-glycans such as asialo-α1-acid glycoprotein (asialo-AGP) to which STL bound weakly (**B20**) (Figure 7).

Only WGA showed extensive, but rather selective binding toward *O*-glycans (not available in Figure 3 except core 1, **911**); i.e., strong binding was found to core 6 (GlcNAcβ1-6GalNAcα1-PAA; **C16**), core 4 (GlcNAcβ1-3(GlcNAcβ1-6)GalNAcα1-PAA; **C14**), and core 2 {Galβ1-3(GlcNAcβ1-6)GalNAcα1-PAA; **C12**}. WGA apparently recognizes GlcNAcβ1-6-terminated *O*-glycans, because no binding was observed to core 1 (Galβ1-3GalNAcα1-PAA; **C11**) and core 8 (Galα1-3Galα1-PAA; **C17**) disaccharides and only weak binding to core 3 (GlcNAcβ1-3GalNAcα1-PAA; **C13**). WGA also bound to Tn antigen (GalNAcα-PAA; **C10**) and its anomer GalNAcβ-PAA (**B15**), but to a much lesser extent compared with GlcNAcβ-PAA (**B23**) and its 6-sulfo form ([6S]GlcNAcβ-PAA; **B24**). WGA bound strongly to a glycolipid-type, Forssman disaccharide (GalNAcα1-3GalNAcβ-PAA; **C15**; **717** in Figure 3).

#### 2.5.2. Binding Features to Glycoproteins

With regard to glycoprotein ligands, Cy3-labeled WGA showed extensive binding to sialylated glycoproteins, though the signals were much stronger for FET (**B1**) and AGP (**B21**) than transferin (TF; **B3**) and thyroglobulin (TG; **B4**). However, DSA and UDA bound almost exclusively to TG (**B4**). This may reflect the difference in sialylation; FET contains sialylated triantennary *N*-glycans as the main components and AGP contains sialylated tri and tetraantennary *N*-glycans, while TF is composed of sialylated biantennary and TG contains high-mannose-type *N*-glycans in addition to biantennary complex-type and hybrid-type *N*-glycans. DSA, PWM, and UDA binding to TG (**B4**) may in part be attributed to binding to high-mannose-type *N*-glycans, which is consistent with FAC results. Other lectins (LEL and STL) also bound to TG (**B4**) but to a much lesser extent. Although these lectins did not show significant affinity for sialylated glycans in FAC, the observed binding to the glycoconjugate microarray is attributed to a clustering effect involving GlcNAc and/or acetyl group of Neu5Ac, a major component of sialic acid.

After desialylation, DSA showed an apparently increased affinity for asialo-FET (**B19**), asialo-AGP (**B20**), and slightly for asialo-TF (**B21**). Conversely, the strong binding observed for TG was greatly reduced (asialo-TG; **B22**). It seems that a common feature of all of the chitin-binding lectins investigated here is that binding to TG was substantially reduced after sialidase treatment. Both WGA and UDA showed significant affinity for these desialylated glycoproteins, though the latter showed much stronger binding to asialo-TG (**B22**). However, LEL, PWM, and STL showed weak binding to asialo-AGP (**B20**), asialo-TF (**B21**), and asialo-TG (**B22**).

UDA showed relatively low, but significant affinity for mannose polymers, such as mannan derived from *Streptococcus aureus* (SA; **D21**) and *Candida albicans* (CA; **D22**), which contrasts with other chitin-binding lectins. This may be related to the FAC result that UDA shows extensive affinity for high-mannose-type *N*-glycans. Indeed, UDA showed strong binding to invertase (Inver; **C9**) and ovalbumin (OVA; **C5**), which have high-mannose-type *N*-glycans as major components.

All of the lectins tested in this work showed significant affinity for ovomucoid (OVM; **C4**) to various extents, which consists of agalactosylated and, thus, GlcNAc-exposed, complex-type *N*-glycans. Particularly strong binding was observed for DSA, which could be attributed to the presence of partially galactosylated multi-antennary, complex-type *N*-glycans, which DSA recognizes preferentially. The presence of bisecting GlcNAc in OVM may explain the strong binding of WGA.

Thus, the interpretation of glycoconjugate microarray, mimicking cell surface probing with these lectins, is not simple. Possible reasons for discrepancies between the results of FAC and glycoconjugate microarray include the cluster effect evident in the latter method. Lectin immobilization may also alter binding properties in FAC as well as in lectin microarray described below. Moreover, it should also be noted that any array platform is basically different from FAC in that the former is a “competitive platform” with a panel of immobilized glycans or lectins, which are reacted in a competitive manner with labeled probes; e.g., Cy3-lectins and glycoproteins. On the other hand, basic specificities of lectins are obtained by FAC using structure-defined purified glycans without any effect of carrier protein and with less effect of clustering of glycans (they are maximally diluted). In this regard, it should also be noted that native glycoproteins consist of considerably heterogeneous glycoforms [75].

### 2.6. Clustering Effect of Sialic Acid; Binding Features on a Lectin Microarray

All six chitin-binding lectins bound to sialylated glycoproteins in the glycoconjugate microarray (Figure 7), while they showed no apparent affinity for sialylated oligosaccharides in the FAC assay (Figure 4). This discrepancy is explained, at least in part, by the glycoside-clustering effect of the acetyl group of sialic acid (Neu5Ac). To examine this idea, binding features were compared on the lectin microarray platform using FET as a model sialoglycoprotein both before and after sialidase treatment.

**Figure 8:**
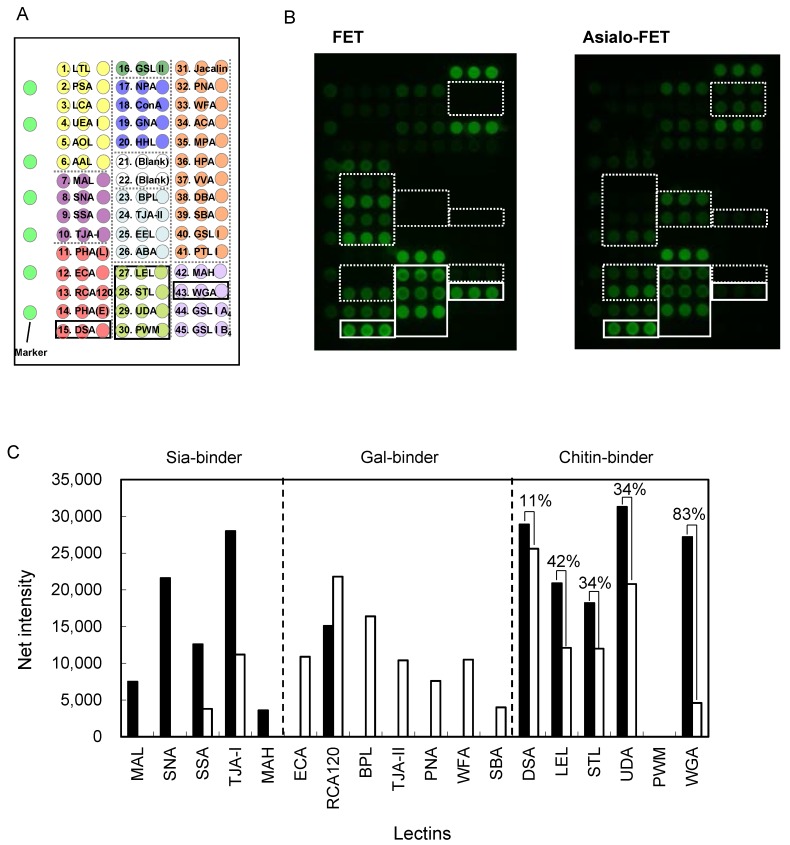
Lectin microarray analysis to observe the effect desialylation of a serum glycoprotein, fetuin (FET). Spot pattern of the lectin microarray; 43 lectins containing the six chitin-binding lectins were spotted on a glass slide, where different colors represent different glycan-binding specificities (**A**); Signal patterns obtained for FET and asialo-FET (**B**); Regions of chitin-binding lectins are indicated with boxes with solid lines and regions lectins showing altered signals by sialidase treatment are indicated with dotted lines. Bar graph representation of signal intensities of relevant lectins both before (closed bars) and after (open bars) sialidase treatment (**C**). Values above bars in the chitin-binding lectins show % reduction in the signal intensities after sialidase treatment.

As shown in Figure 8, signal intensities of a series of Sia-binding lectins (MAL, SNA, SSA, TJA-I, and MAH) were drastically decreased after desialylation. Conversely, signal intensities of Gal-binding lectins (ECA, BPL, TJA-II, PNA WFA, and SBA) became much stronger after the sialidase treatment, with the exception of RCA120. It is known that RCA120 does not completely lose affinity for Siaα2-6Gal [9], which is a major linkage of sialic acid in FET [76,77].

Among the six chitin-binding lectins, signal intensities of DSA, LEL, STL, and UDA were reduced for asialo-FET by approximately 11%, 42%, 34%, and 34%, respectively. However, WGA showed as much as 83% reduction in the observed signal after desialylation. Thus, the effect of sialic acid clustering is significant, but rather modest for most of the chitin-binding lectins under conditions where lectins are immobilized and diluted sialoglycoprotein, such as in FET. However, WGA showed enhanced affinity for FET compared with asialo-FET, similar to that in the glycoconjugate microarray. Therefore, sialic acid clustering is sufficient for WGA recognition at a glycoprotein level (triantennary *N*-glycans in FET), but not at a liberated glycan level as is the case in FAC analysis.

### 2.7. Future Engineering of Chitin-Binding Proteins as Glycan Probes

Historically, plant lectins have been used for analysis and separation of various glycans, which are mainly of animal origin. Among them, however, chitin-binding lectins have been used to detect and inhibit parasites or viruses, which recognize chito-oligosaccharides as target molecules [78,79]. They have also been shown to be useful probes for cell surface glycan structures containing poly-LacNAc [24,61,62]. A recent study supports the idea that various lectins, including chitin-binding lectins, can prevent viral infection in vivo [80].

In this study, six representative chitin-binding lectins belonging to the hevein family were investigated in detail for their sugar-binding properties (for summary, see Table 2). However, there are many other chitin-binding lectins, which do not belong to the hevein family [81]. They include LysM (lysin motif), which was initially found in several bacterial autolysin proteins and is often associated with chitinase as a carbohydrate-binding module [82]. Interestingly, LysM also consists of a small number (approximately 50) of amino acids, including evolutionarily conserved disulfide bonds. It seems, however, that binding features to chito-oligosaccharides are fairly different between the two families [81].

Considering both compact and stable structures, chitin-binding lectins are good targets for protein engineering aimed at improving specific properties, which natural lectins lack. More diverse oligosaccharide specificity will be generated by recently developed molecular engineering strategies [83,84]. Alternatively, tandem-repeat type lectins, such as WGA and UDA, may improve their performance both in terms of binding affinity and specificity by selecting one of their repeating CRDs resulting in a “homogenous” tandem-repeat. It is widely believed that individual CRDs, after duplication in the course of evolution, diverge from each other and, thus, their specificities have also diverged. In this context, it is important to identify a CRD responsible for the desired specificity, e.g., affinity for hybrid-type *N*-glycans containing bisecting GlcNAc in WGA. So far, however, such an approach has never been made with chitin-binding lectins. Future studies necessary for this approach include practical application of naturally and artificially produced chitin-binding lectins in various fields, e.g., medical, material and food sciences. Using the information obtained in these studies will generate more precise and systematic knowledge for lectin utilization in complex life systems.

## 3. Materials and Methods

### 3.1. Materials

*N*-hydroxysuccinimide (NHS)-activated Sepharose 4 Fast Flow was purchased from GE Healthcare (Little Chalfont, Buckinghamshire, UK). All chemical reagents used in this study were of analytical grade. FET from fetal calf serum (48,400 Da) was purchased from Sigma (St. Louis, MO, USA).

### 3.2. Oligosaccharides

*p*NP glycosides Galβ1-4GlcNAcβ (LacNAc-β-*p*NP), GlcNAcβ1-4GlcNAcβ1-4GlcNAcβ1-4GlcNAcβ1 (chitotetraose-β-*p*NP), and GlcNAcβ1-4GlcNAcβ1-4GlcNAcβ1-4GlcNAcβ1-4GlcNAcβ1 (chitopentaose-β-*p*NP), were obtained from Toronto Research Chemicals, Inc. (North York, ON, Canada).

Structure-defined, purified standard PA-oligosaccharides used in this study are shown in Figure 3. PA-*N*-glycans numbered **001**–**014**, **103**, **105**, **107**, **108**, **307**, **313**, **314**, **323**, **405**, **410**, **418**–**420**, **501**–**504**, and **506** were purchased from Takara Bio, Inc. (Kyoto, Japan), **304**, **403**, and **404** were obtained from J-OIL MILLS. Inc. (Tokyo, Japan), and other PA-*N*-glycans were obtained from Seikagaku Co. The non-labeled glycans (**601** and **602**) were obtained from Dextra Laboratories, Ltd. (Reading, UK) and labeled with 2-aminopyridine with a GlycoTAG (Takara Bio, Inc.). The derived PA-*N*-glycans were purified and quantified as described previously [85].

Glycolipid and other types of glycans, **701**–**703**, **705**–**713**, **715**–**718**, **720**, **721**, **724**, **726** and **728**–**731**, were obtained as their PA-derivatives from Takara Bio, Inc. or as non-labeled glycans which were pyridylaminated: **727** from Funakoshi Co., **704**, **733**, and **734** from Dextra Laboratories, Ltd., **725**, **909**, **910**, and **911** from Calbiochem, and **906** and **907** from Seikagaku Co. Oligo-*N*-acetyllactosamines **901**–**903** and **905** and milk oligosaccharides **722**, **723**, **732**, and **735**–**739** were generous gifts from K. Yoshida (Seikagaku Co., Tokyo, Japan) and T. Urashima (Obihiro University of Agriculture and Veterinary Medicine, Obihiro, Japan), respectively.

### 3.3. Preparation of Lectin Columns

Lectin columns were prepared by coupling to NHS-activated Sepharose 4 Fast Flow according to the manufacturer’s instructions. DSA, PWM, UDA, and WGA were obtained from J-OIL MILLS. Inc., and LEL and STL were from Vector Lab. (Burlingame, CA, USA). They were dissolved in 100 mM NaHCO_3_ buffer (pH 8.3) containing 0.5 M NaCl for coupling, and excess NHS groups were deactivated by 0.5 M monoethanolamine (pH 8.3) containing 0.5 M NaCl. After extensive washing with 0.1 M CH_3_COONa buffer (pH 4.0) and then with 0.1 M NaHCO_3_ buffer (pH 8.3) containing 0.5 M NaCl, the resultant lectin-Sepharose was suspended in 10 mM Tris-HCl (pH 7.4) containing 0.8% (*w*/*v*) NaCl (TBS). For FAC analysis, the slurry of each lectin-Sepharose was packed into a miniature column (inner diameter, 2 mm; length, 10 mm; bed volume, 31.4 μL; Shimadzu Co., Kyoto, Japan) as described previously [9,48,52]. The amount of uncoupled protein in the above wash fraction was determined by the Bradford method, and hereby, the amount of immobilized protein was estimated.

### 3.4. Frontal Affinity Chromatography (FAC)

FAC was performed using an automated system, FAC-1 or FAC-T, as described previously [9,48,52]. Briefly, the above lectin-immobilized columns were connected to the FAC-1 or FAC-T and were equilibrated with TBS. The flow rate and column temperature were kept at 0.125 mL/min and 25 °C, respectively. After equilibration of the columns, either excess volume of PA or *p*NP-oligosaccharides dissolved in TBS were successively injected into each of the lectin columns. Elution of PA-oligosaccharides (2.5 or 5.0 nM) was monitored by fluorescence (excitation and emission wavelengths of 310 and 380 nm, respectively), while that of *p*NP-oligosaccharides (2–60 μM) was monitored by UV adsorption (280 nm). The volume of the elution front (*V*) of each oligosaccharide was calculated as described previously [46,47,48]. *V*_0_ was determined for an appropriate reference substance; i.e., Manα1-6(Manα1-2)Manβ1-4GlcNAcβ1-4GlcNAc-PA (**003**) for DSA, LEL, STL, and WGA, Galβ1-4Glc-PA (**701**) for UDA, and **003** or Galβ1-3GalNAcβ1-4Galβ1-4Glc-PA (**703**) for PWM. Hence, retardation of the elution front of each oligosaccharide, i.e., *V* − *V*_0_, was used for determination of *K*_d_ values. Since [A]_0_ is, in most cases, much smaller than *K*_d_, the equation can be simplified to be *K*_d_ = *B*_t_/(*V* − *V*_0_), where *K*_d_ value is no longer dependent on [A]_0_.

It is often useful to discuss lectin-oligosaccharide interactions in terms of an affinity constant, *K*_a_ (1/*K*_d_), instead of *K*_d_. When binding affinity of a particular glycan was too high to measure *V* − *V*_0_, we determined its *K*_d_ (or *K*_a_) value using another column with an appropriately lowered lectin concentration. This was applied in the cases of DSA (6.0 and 1.5 mg/mL), UDA (6.0, 3.0, 1.0, and 0.3 mg/mL), WGA (7.0, 2.0, and 0.5 mg/mL), and LEL (6.0 and 0.5 mg/mL). To combine data from the two columns, a ratio between them was determined using *V* − *V*_0_ values of a reference glycan, which showed significant retardation in both columns.

### 3.5. Concentration-Dependent Analysis

For the determination of *B*_t_ and *K*_d_ values for each lectin column, an appropriate *p*NP-saccharide was diluted to various concentrations (2–100 μM) and was used for concentration-dependent analysis. Using the resulting *V* values, Woolf-Hofstee-type plots, i.e., (*V* − *V*_0_) vs. (*V* − *V*_0_)[A]_0_, were constructed to determine *B*_t_ and *K*_d_ values from the intercept and the slope of the fitted curves, respectively, as described previously [9].

### 3.6. Glycoconjugate-Microarray Analysis

To validate the binding specificity data obtained by FAC and observe the effect of clustering on the immobilized glycans, glycoconjugate microarray analysis was performed [49]. Briefly, 60 μL of Cy3-labeled lectins (at a final concentration of 4.0 μg/mL) used for FAC was applied to the glycoconjugate microarray (ver. 4.2). After incubation for interaction at 20 °C overnight, wells on the glass slide were rinsed twice with the probing buffer (Tris-buffered saline, pH 7.4 containing 1% Triton X-100, 1 mM CaCl_2_, and 1 mM MnCl_2_) for the microarray and fluorescence images of the microarray were acquired using an evanescent-field activated fluorescence scanner, GlycoStation Reader™ 1200 (GlycoTechnica Ltd., Yokohama, Japan). Scanning conditions were as follows: exposure time, 199 ms; detection sensitivities, gain 115 for DSA, LEL, STL, and UDA, gain 125 for PWM, and gain 110 for STL.

### 3.7. Lectin Microarray Analysis

To investigate the clustering effect for sialic acid (Neu5Ac), another interaction analysis was performed between immobilized lectins and a sialoglycoprotein, FET, in a lectin microarray platform. Asialo-FET was prepared by digestion of FET with sialidase A (Prozyme Inc., San Leandro, CA, USA) according to the protocol provided by the manufacturer. For comparison, solutions of FET and asialo-FET were prepared at 2.0 μg/mL and were applied to a lectin microarray for analysis as described previously [9]. Scanning conditions were as follows: exposure time, 199 ms; detection sensitivities, gain 90 and 80 for FET and asialo-FET, respectively. Data were analyzed with the Array Pro analyzer ver. 4.5 (Media Cybernetics, Rockville, MD, USA) and are represented by bar graphs in terms of net signal intensities.

## 4. Conclusions

In the present study, various novel features of chitin-binding lectins became evident (Table 2). It also became clear that results of FAC and glycoconjugate microarray are not necessarily consistent, and the interpretation of glycoconjugate microarray is not simple. Nevertheless, data derived by the present quantitative FAC analysis using structure-defined standard glycans provide key information to solve complex phenomena of biological systems, which include studies using these lectins in histochemical and cell separation studies. Currently, it is not known whether the described characteristics are attributed to differences in overall structural architectures (i.e., proto, chimera, or tandem-repeat) or in amino-acid sequences in CRDs. Detailed structural analysis is necessary to define the sugar-binding specificity of each CRD. Construction of tandem-repeat type homogenous hevein-like domains should lead to the development of a series of artificial chitin-binding lectins with much stronger avidity and much higher specificity.

## Figures and Tables

**Table 1:** Specifications of lectin columns used for FAC analysis.

Lectin Name	Origin	Organ	Immobilized (mg/mL Resin)	*B*_t_ (nmol)	*r*^2 a^	Glycan-*p*NP Used for Specification	*K*_d _ (μM)
**DSA**	*Datura stramonium*	Seed	6.0	1.12	1.00	chitotetraose-β-*p*NP	33
**LEL**	*Solanum lycopersicum*	Fruit	6.0	0.74	0.99	LacNAc-β-*p*NP	7.9
			0.5	0.11	0.96	chitopentaose-β-*p*NP^ b^	4.6
**PWM**	*Phytolacca Americana*	Plant root	4.6	1.58	0.96	chitopentaose-β-*p*NP	42
**STL**	*Solanum tuberosum*	Plant tuber	3.0	1.10	1.00	chitopentaose-β-*p*NP	31
**UDA**	*Urtica dioica*	Rhizome	6.0	6.10	0.99	LacNAc-β-*p*NP	48
**WGA**	*Triticum vulgaris*	Germ	7.0	5.55	0.99	LacNAc-β-*p*NP	57

^a^ Reliability of lines obtained as a result of Woolf-Hofstee-type plot. FAC: frontal affinity chromatography; DSA: *Datura stramonium* agglutinin; LEL: *Lycopersicon esculentum* lectin; PWM: pokeweed mitogen; STL: *Solanum tuberosum* lectin; UDA: *Urtica dioica* agglutinin; WGA: wheat germ agglutinin. ^b ^For the determination of strong binding between LEL and chitotetraose-PA, another column was prepared with a lower *B*_t_, for which determination chitopentaose-β-*p*NP was used.

**Table 2:** Summary of sugar-binding features of the chitin-binding lectins investigated in the present work

Lectin	DSA ^a^	LEL ^b^	PWM ^c^	STL ^d^	UDA ^e^	WGA ^f^
Common features: Binding to chito-oligo						
chitotriose-PA (**906**)	N.D.	4.6 μM	N.D.	N.D.	57 μM	4.7 μM
chitotetraose-PA (**907**)	43 μM	0.64 μM	53 μM	12 μM	3.8 μM	4.1 μM
Binding to LNH/LN*n*H						
LN*n*H (type II+ type II; **733**)	43 μM	6.7 μM	130 μM	130 μM	29 μM	93 μM
LNH (type I + type II; **734**)	19 μM	3.5 μM	93 μM	220 μM	35 μM	110 μM
Unique features:						
3 other best PA-oligosaccharides	4.0 μM (**323**)	2.9 μM (**905**)	150 μM (**004**)	LacdiNAc-PAA (Glycoconjugate microarray)	3.7 μM (**016**)	19 μM (**053**)
	4.1 μM (**418**)	10 μM (**903**)	300 μM (**005**)		4.0 μM (**014**)	19 μM (**058**)
	5.2 μM (**420**)	39 μM (**902**)	300 μM (**007**)		5.5 μM (**011**)	20 μM (**051**)

^a^ DSA shows affinity to highly branched* N*-glycans containing intact type II LacNAc, e.g., **323**, **418** and **420**; ^b ^LEL shows substantial affinity to repeated LacNAc structures, e.g., **902**, **903 **and **905**; ^c^ PWM shows relatively weak but significant affinity to a few members of high-mannose-type *N*-glycans, i.e., **004**, **005** and **007**.; ^d^ STL shows the simplest binding profile among the investigated lectins in FAC, while showing rather selective binding to LacdiNAc-PAA in glycoconjugate microarray; ^e^ UDA shows extensive binding to high-mannose-type *N*-glycans with the structural unit Manα1-3(Manα1-6)Manα1-6Manβ, e.g., **011**, **014** and **016**; ^f^ WGA shows extensive binding to not only GlcNAc-containing glycoconjugates but also those having Neu5Ac clusters. WGA also shows selective binding to hybrid-type *N*-glycans having bisecting GlcNAc, e.g., **051**, **053** and **058**. N.D.: Not Detectable.

## Supplementary Materials

Supplementary materials are available online at www.mdpi.com/1422-0067/18/6/1160/s1.

## Abbreviations

AGP	α1-acid glycoprotein
BSA	Bovine serum albumin
BSM	Bovine submaxillary gland mucin
CRD	Carbohydrate-recognition domain
CSA	Chondroitin sulfate A
CSB	Chondroitin sulfate B
DSA	*Datura stramonium* agglutinin
FAC	Frontal affinity chromatography
FET	Fetuin
GnT	GlcNAc transferase
GSL	*Griffonia simplicifolia* lectin
HA	Hyaluronic acid
HP	Heparin
HS	Heparin sulfate
Inver	Invertase
KS	Keratin sulfate
LDN	LacdiNAc
LEL	*Lycopersicon esculentum* lectin
LN	LacNAc
OVA	Ovalbumin
OVM	Ovomucoid
PA	Pyridylaminated
*p*NP	*p*-nitrophenyl
PWM	*Phytolacca americana* (pokeweed) mitogen
RCA	*Ricinus communis* agglutinin
STL	*Solanum tuberosum* lectin
TF	Transferrin
TG	Thyroglobulin
UDA	*Urtica dioica* agglutinin
WGA	*Triticum vulgaris* (wheat germ) agglutinin
