# Supplementary material for: Sugar-Binding Profiles of Chitin-Binding Lectins from the Hevein Family: A Comprehensive Study
Source: Int J Mol Sci. 2017 May 30;18(6):1160. doi: 10.3390/ijms18061160 (PMC5485984; doi:10.3390/ijms18061160)
Supplement: Supplementary file 1 [file ijms-18-01160-s001.zip › ijms-196287-spl-proofback/Itakura_TableS1.pdf]

Supplementary table 1-1. **Frontal affinity chromatograph data of six lectins.**

lectin	DSA		LEL		PWM		STL		UDA		WGA	
glycans	$V-V_0$ (μl)	K_d (μM)	$V-V_0$ (μl)	K_d (μM)	$V-V_0$ (μl)	K_d (μM)	$V-V_0$ (μl)	K_d (μM)	$V-V_0$ (μl)	K_d (μM)	$V-V_0$ (μl)	K_d (μM)
001	0	N.D.[a]	0	N.D.	0	N.D.	0	N.D.	32.3	190	0	N.D.
002	0	N.D.	8.3	89	0	N.D.	0	N.D.	26.3	230	19.7	280
003	0	N.D.	0	N.D.	0	N.D.	0	N.D.	38.9	160	0	N.D.
004	0	N.D.	0	N.D.	10.8	150	0	N.D.	425.4	14	0	N.D.
005	0	N.D.	0	N.D.	5.2	300	0	N.D.	502.1	12	0	N.D.
006	0	N.D.	0	N.D.	0	N.D.	0	N.D.	543.6	11	0	N.D.
007	0	N.D.	0	N.D.	5.2	300	0	N.D.	727.4	8.4	0	N.D.
008	0	N.D.	0	N.D.	0	N.D.	0	N.D.	709.8	8.6	0	N.D.
009	0	N.D.	0	N.D.	0	N.D.	0	N.D.	675.8	9.0	0	N.D.
010	18.6	60	0	N.D.	0	N.D.	0	N.D.	761.4	8.0	7.3	760
011	20.1	56	0	N.D.	0	N.D.	0	N.D.	1117.5	5.5	6.3	880
012	0	N.D.	0	N.D.	0	N.D.	0	N.D.	1017.4	6.0	0	N.D.
013	0	N.D.	0	N.D.	0	N.D.	0	N.D.	938.4	6.5	0	N.D.
014	0	N.D.	0	N.D.	0	N.D.	0	N.D.	1528.8	4.0	0	N.D.
015	0	N.D.	0	N.D.	0	N.D.	0	N.D.	29.6	210	0	N.D.
016	0	N.D.	0	N.D.	0	N.D.	0	N.D.	1665.9	3.7	0	N.D.
017	0	N.D.	0	N.D.	0	N.D.	0	N.D.	0	N.D.	5.1	1100
052	0	N.D.	0	N.D.	0	N.D.	0	N.D.	0	N.D.	188.3	30
101	0	N.D.	0	N.D.	0	N.D.	0	N.D.	0	N.D.	9.9	560
102	0	N.D.	0	N.D.	0	N.D.	0	N.D.	13.6	450	0	N.D.
103	0	N.D.	0	N.D.	0	N.D.	0	N.D.	0	N.D.	0	N.D.
104	0	N.D.	0	N.D.	0	N.D.	0	N.D.	0	N.D.	5.4	1000
105	0	N.D.	0	N.D.	0	N.D.	0	N.D.	0	N.D.	0	N.D.
107	0	N.D.	0	N.D.	0	N.D.	0	N.D.	0	N.D.	0	N.D.
108	0	N.D.	0	N.D.	0	N.D.	0	N.D.	0	N.D.	4.1	1400
201	0	N.D.	0	N.D.	0	N.D.	0	N.D.	0	N.D.	0	N.D.
202	0	N.D.	0	N.D.	0	N.D.	0	N.D.	0	N.D.	0	N.D.
203	0	N.D.	0	N.D.	0	N.D.	0	N.D.	0	N.D.	4.5	1200
301	0	N.D.	0	N.D.	0	N.D.	0	N.D.	0	N.D.	0	N.D.
302	0	N.D.	0	N.D.	0	N.D.	0	N.D.	32.1	190	0	N.D.
304	0	N.D.	0	N.D.	0	N.D.	0	N.D.	0	N.D.	0	N.D.
305	0	N.D.	0	N.D.	0	N.D.	0	N.D.	0	N.D.	4.6	1200
307	0	N.D.	0	N.D.	0	N.D.	5.3	210	8.9	690	0	N.D.
308	0	N.D.	0	N.D.	0	N.D.	0	N.D.	0	N.D.	0	N.D.
313	53.6	21	0	N.D.	0	N.D.	0	N.D.	0	N.D.	0	N.D.
314	0	N.D.	0	N.D.	0	N.D.	0	N.D.	0	N.D.	0	N.D.
323	281.0	4.0	0	N.D.	0	N.D.	0	N.D.	0	N.D.	0	N.D.
401	0	N.D.	0	N.D.	0	N.D.	0	N.D.	0	N.D.	0	N.D.
402	0	N.D.	0	N.D.	0	N.D.	0	N.D.	31.6	190	0	N.D.
403	0	N.D.	0	N.D.	0	N.D.	0	N.D.	0	N.D.	0	N.D.
404	0	N.D.	0	N.D.	0	N.D.	0	N.D.	0	N.D.	9	620
405	0	N.D.	0	N.D.	0	N.D.	0	N.D.	0	N.D.	0	N.D.
406	0	N.D.	0	N.D.	0	N.D.	0	N.D.	0	N.D.	0	N.D.
410	50.3	22	0	N.D.	0	N.D.	0	N.D.	0	N.D.	0	N.D.
418	273.1	4.1	0	N.D.	0	N.D.	0	N.D.	0	N.D.	0	N.D.
419	0	N.D.	0	N.D.	0	N.D.	0	N.D.	0	N.D.	0	N.D.
420	214.5	5.2	0	N.D.	0	N.D.	0	N.D.	0	N.D.	0	N.D.
501	0	N.D.	0	N.D.	0	N.D.	0	N.D.	0	N.D.	0	N.D.
502	0	N.D.	0	N.D.	0	N.D.	0	N.D.	0	N.D.	0	N.D.
503	0	N.D.	0	N.D.	0	N.D.	0	N.D.	0	N.D.	0	N.D.
504	0	N.D.	0	N.D.	0	N.D.	0	N.D.	0	N.D.	0	N.D.
506	0	N.D.	0	N.D.	0	N.D.	0	N.D.	0	N.D.	6.0	930
601	0	N.D.	0	N.D.	0	N.D.	0	N.D.	0	N.D.	0	N.D.
602	0	N.D.	0	N.D.	0	N.D.	0	N.D.	0	N.D.	0	N.D.

Supplementary table 1-2. **Frontal affinity chromatograph data of six lectins.**

Lectin	DSA		LEL		PWM		STL		UDA		WGA	
Glycans	$V\text{-}V_0$ (µl)	K_d (µM)	$V\text{-}V_0$ (µl)	K_d (µM)	$V\text{-}V_0$ (µl)	K_d (µM)	$V\text{-}V_0$ (µl)	K_d (µM)	$V\text{-}V_0$ (µl)	K_d (µM)	$V\text{-}V_0$ (µl)	K_d (µM)
701	35.8	31	0	N.D.	0	N.D.	0	N.D.	0	N.D.	0	N.D.
702	0	N.D.	0	N.D.	0	N.D.	0	N.D.	0	N.D.	0	N.D.
703	0	N.D.	0	N.D.	0	N.D.	0	N.D.	0	N.D.	0	N.D.
704	12.6	89	0	N.D.	0	N.D.	0	N.D.	0	N.D.	0	N.D.
705	0	N.D.	0	N.D.	0	N.D.	0	N.D.	0	N.D.	5.3	1000
706	0	N.D.	0	N.D.	0	N.D.	0	N.D.	0	N.D.	0	N.D.
707	0	N.D.	0	N.D.	0	N.D.	0	N.D.	0	N.D.	0	N.D.
708	12.3	91	0	N.D.	0	N.D.	0	N.D.	0	N.D.	0	N.D.
709	0	N.D.	0	N.D.	0	N.D.	0	N.D.	0	N.D.	0	N.D.
710	30.1	37	0	N.D.	0	N.D.	0	N.D.	0	N.D.	5.3	1000
711	0	N.D.	0	N.D.	0	N.D.	0	N.D.	0	N.D.	0	N.D.
712	0	N.D.	0	N.D.	0	N.D.	0	N.D.	0	N.D.	4.9	1100
713	0	N.D.	0	N.D.	0	N.D.	0	N.D.	0	N.D.	0	N.D.
715	0	N.D.	0	N.D.	0	N.D.	0	N.D.	0	N.D.	0	N.D.
716	0	N.D.	0	N.D.	0	N.D.	0	N.D.	0	N.D.	0	N.D.
717	0	N.D.	0	N.D.	0	N.D.	0	N.D.	0	N.D.	9.4	590
718	0	N.D.	0	N.D.	0	N.D.	0	N.D.	0	N.D.	0	N.D.
719	13.5	83	0	N.D.	0	N.D.	0	N.D.	12.6	480	7.7	720
720	0	N.D.	0	N.D.	0	N.D.	0	N.D.	0	N.D.	4.4	1300
721	20.9	54	0	N.D.	0	N.D.	0	N.D.	0	N.D.	0	N.D.
722	0	N.D.	0	N.D.	0	N.D.	0	N.D.	0	N.D.	0	N.D.
723	0	N.D.	0	N.D.	0	N.D.	0	N.D.	0	N.D.	0	N.D.
724	12.3	91	0	N.D.	0	N.D.	0	N.D.	4.3	1400	5.5	1000
725	16.3	69	0	N.D.	0	N.D.	0	N.D.	5.1	1200	8.0	690
726	0	N.D.	0	N.D.	0	N.D.	0	N.D.	0	N.D.	0	N.D.
727	14.9	75	0	N.D.	0	N.D.	0	N.D.	0	N.D.	0	N.D.
728	0	N.D.	0	N.D.	0	N.D.	0	N.D.	0	N.D.	0	N.D.
729	17.2	65	0	N.D.	0	N.D.	0	N.D.	0	N.D.	0	N.D.
730	13.8	81	0	N.D.	0	N.D.	0	N.D.	0	N.D.	0	N.D.
731	16.6	67	0	N.D.	0	N.D.	0	N.D.	0	N.D.	0	N.D.
732	0	N.D.	0	N.D.	0	N.D.	0	N.D.	0	N.D.	0	N.D.
733	25.9	43	110.4	6.7	12.4	130	8.2	130	208.3	29	59.4	93
734	59.0	19	208.9	3.5	17.0	93	5.0	220	174.9	35	52.9	110
735	15.2	74	0	N.D.	0	N.D.	0	N.D.	0	N.D.	0	N.D.
736	18.0	62	0	N.D.	0	N.D.	0	N.D.	0	N.D.	0	N.D.
737	0	N.D.	0	N.D.	0	N.D.	0	N.D.	0	N.D.	0	N.D.
738	0	N.D.	0	N.D.	0	N.D.	0	N.D.	0	N.D.	0	N.D.
739	0	N.D.	0	N.D.	0	N.D.	0	N.D.	0	N.D.	0	N.D.
901	12.9	87	0	N.D.	0	N.D.	0	N.D.	0	N.D.	0	N.D.
902	23.5	48	19.1	39	0	N.D.	0	N.D.	4.6	1300	5.6	990
903	25.8	43	71.7	10	0	N.D.	0	N.D.	13.2	460	10.1	550
905	65.3	17	256.7	2.9	0	N.D.	0	N.D.	62.5	98	21.5	260
906	0	N.D.	161.1	4.6	0	N.D.	0	N.D.	107.0	57	1189.9	4.7
907	26.2	43	172.5	0.64	29.9	53	90.1	12	1586.8	3.8	1357.9	4.1
908	42.7	26	0	N.D.	0	N.D.	0	N.D.	0	N.D.	4.7	1200
909	18.0	62	0	N.D.	0	N.D.	0	N.D.	0	N.D.	0	N.D.
910	0	N.D.	0	N.D.	0	N.D.	0	N.D.	0	N.D.	0	N.D.
911	0	N.D.	0	N.D.	0	N.D.	0	N.D.	0	N.D.	0	N.D.
913	0	N.D.	0	N.D.	0	N.D.	0	N.D.	0	N.D.	4.1	1400
914	14.3	78	0	N.D.	0	N.D.	0	N.D.	0	N.D.	0	N.D.
915	0	N.D.	0	N.D.	0	N.D.	0	N.D.	0	N.D.	0	N.D.
918	12.3	91	0	N.D.	0	N.D.	0	N.D.	0	N.D.	0	N.D.
919	0	N.D.	0	N.D.	0	N.D.	0	N.D.	0	N.D.	0	N.D.
920	18.0	62	0	N.D.	0	N.D.	0	N.D.	0	N.D.	0	N.D.
921	0	N.D.	0	N.D.	0	N.D.	0	N.D.	0	N.D.	0	N.D.
927	42.7	26	0	N.D.	0	N.D.	0	N.D.	0	N.D.	0	N.D.
928	32.4	35	0	N.D.	0	N.D.	0	N.D.	0	N.D.	0	N.D.
929	37.0	30	0	N.D.	0	N.D.	0	N.D.	0	N.D.	0	N.D.
930	35.5	32	0	N.D.	0	N.D.	0	N.D.	0	N.D.	0	N.D.
936	0	N.D.	21.3	35	0	N.D.	0	N.D.	31.4	190	14.8	380
937	0	N.D.	33.8	22	0	N.D.	0	N.D.	36.3	170	10.5	530
051	-[b]	-	-	-	-	-	-	-	-	-	271.7	20
053	-	-	-	-	-	-	-	-	-	-	286.4	19
055	-	-	-	-	-	-	-	-	-	-	169.5	33
056	-	-	-	-	-	-	-	-	-	-	0	N.D.
057	-	-	-	-	-	-	-	-	-	-	0	N.D.
058	-	-	-	-	-	-	-	-	-	-	292.4	19
205	-	-	-	-	-	-	-	-	-	-	0	N.D.
310	-	-	-	-	-	-	-	-	-	-	32.7	170
311	-	-	-	-	-	-	-	-	-	-	18.9	290

[a] “N.D.” mean “not detected”. [b] “-” is no date.
