# Supplementary figures and images for: Sugar-Binding Profiles of Chitin-Binding Lectins from the Hevein Family: A Comprehensive Study
Source: Int J Mol Sci. 2017 May 30;18(6):1160. doi: 10.3390/ijms18061160 (PMC5485984; doi:10.3390/ijms18061160)

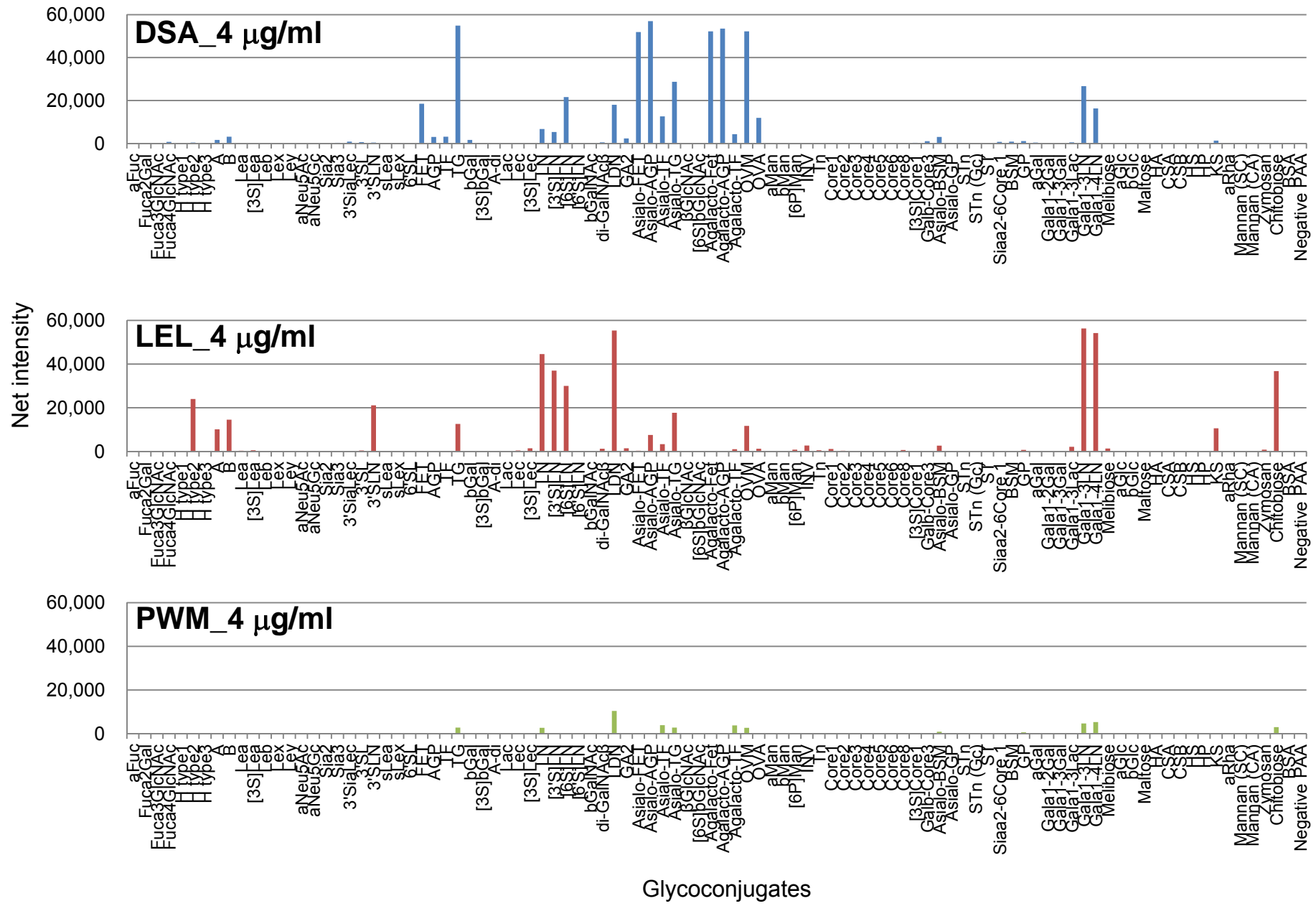

Supplement: Supplementary file 1 [file ijms-18-01160-s001.zip › ijms-196287-spl-proofback/Itakura_FigS1.pdf]
